# On‐Demand, Direct Printing of Nanodiamonds at the Quantum Level

**DOI:** 10.1002/advs.202103598

**Published:** 2021-12-23

**Authors:** Zhaoyi Xu, Lingzhi Wang, Xiao Huan, Heekwon Lee, Jihyuk Yang, Zhiwen Zhou, Mojun Chen, Shiqi Hu, Yu Liu, Shien‐Ping Feng, Tongtong Zhang, Feng Xu, Zhiqin Chu, Ji Tae Kim

**Affiliations:** ^1^ Department of Mechanical Engineering The University of Hong Kong Pokfulam Road Hong Kong China; ^2^ Department of Electrical and Electronic Engineering The University of Hong Kong Pokfulam Road Hong Kong China; ^3^ Joint Appointment with School of Biomedical Sciences The University of Hong Kong Pokfulam Road Hong Kong China

**Keywords:** electrohydrodynamic printing, lithography‐free manufacturing, nanodiamonds, nitrogen vacancy centers, quantum nanomaterials

## Abstract

The quantum defects in nanodiamonds, such as nitrogen‐vacancy (NV) centers, are emerging as a promising candidate for nanoscale sensing and imaging, and the controlled placement with respect to target locations is vital to their practical applications. Unfortunately, this prerequisite continues to suffer from coarse positioning accuracy, low throughput, and process complexity. Here, it is reported on direct, on‐demand electrohydrodynamic printing of nanodiamonds containing NV centers with high precision control over quantity and position. After thorough characterizations of the printing conditions, it is shown that the number of printed nanodiamonds can be controlled at will, attaining the single‐particle level precision. This printing approach, therefore, enables positioning NV center arrays with a controlled number directly on the universal substrate without any lithographic process. The approach is expected to pave the way toward new horizons not only for experimental quantum physics but also for the practical implementation of such quantum systems.

## Graphical abstract

Direct printing of nanodiamonds containing nitrogen‐vacancy centers at the single‐particle level is realized with programmed electrohydrodynamic dispensing of nanodiamonds‐laden nanodroplets.

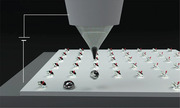

## 1 Introduction

The point defects hosted in diamond crystals, like the nitrogen‐vacancy (NV) centers and other emerging group IV^[^ ^1^ ^]^ ones such as Silicon vacancy centers (SiVs), have emerged as a powerhouse for quantum information processing,^[^ ^2^, ^3^, ^4^, ^5^, ^6^ ^]^ quantum computing,^[^ ^7^, ^8^, ^9^ ^]^ quantum optics,^[^ ^10^, ^11^, ^12^ ^]^ and quantum sensing.^[^ ^13^, ^14^, ^15^ ^]^ As one of the most recognized diamond defects, the NV centers show their unique quantum properties and robustness even at room temperature,^[^ ^16^, ^17^ ^]^ offering a feasible avenue for the realization of quantum technologies. In particular, the NV centers can be hosted in nanoscale diamond particles, namely nanodiamonds, serving as multifunctional nanoagents for many exciting opportunities in diverse fields.^[^ ^18^, ^19^, ^20^ ^]^ For example, NV‐center nanodiamonds have demonstrated their capability as nanoscale probes for magnetometry^[^ ^21^ ^]^ and thermometry,^[^ ^15^, ^22^, ^23^ ^]^ and as biomarkers for tracking dynamic biological processes in living organisms.^[^ ^24^, ^25^ ^]^ Due to the “fragile nature” of quantum systems,^[^ ^26^ ^]^ exploiting their full capabilities necessitates a strategy to manipulate and access them on demand with high precision.

Considerable research has been made to produce and manipulate nanodiamonds containing NV centers. The nanodiamonds can be easily mass produced^[^ ^27^ ^]^ and have become commercially available for years. The introduction of NV centers has been achieved mainly through ion implantations, that is, the use of focused nitrogen,^[^ ^28^ ^]^ helium ion,^[^ ^29^ ^]^ or electron beam^[^ ^30^ ^]^ irradiation with the sub‐micrometer spatial resolution. Nevertheless, the “inhomogeneous” nature of nanodiamonds is brought by their fabrication methods, that is, each individual particle maintaining distinct size, morphology, and surface property.^[^ ^31^ ^]^ This further makes it difficult to realize the envisaged scenario in which the nanodiamonds can be positioned to the targeted locations with high precision.

Several attempts have been devised to improve the compatibility of nanodiamonds with various substrates and circuits.^[^ ^32^, ^33^, ^34^ ^]^ First, stochastic methods based on drop‐casting or spin‐coating provide a simple and cost‐effective route to place NV‐center nanodiamonds on substrates^[^ ^32^ ^]^ but suffer from randomness in the particle positioning. “Pick‐and‐place” methods that use a nano‐manipulator with real‐time observation have been implemented to improve the positional accuracy^[^ ^33^, ^35^ ^]^ and demonstrated exciting progress regarding the near‐field coupling of NV centers to nanophotonic structures. These sophisticated methods, however, challenge to satisfy the required throughput. Although lithographically prepared electrostatic patterns^[^ ^34^, ^36^, ^37^ ^]^ or direct inkjet printing approaches^[^ ^38^, ^39^ ^]^ have recently been utilized for large‐scale integration of NV centers, a universal and flexible manufacturing route is still in great demand for achieving nanoscale accuracy, scalability, cost‐effectiveness, and efficient coupling with a wide range of nanophotonic circuitries.

Here, we develop an electrohydrodynamic (EHD) dispensing method to print nanodiamonds containing NV centers with programmed quantity and position directly on common substrates without the need for any lithography process. The EHD ejection dynamics and suspension stability of nanodiamonds‐laden droplets with sub‐attoliter volume are quantitatively investigated for achieving a high‐precision and high‐fidelity printing process. The results demonstrate sub‐wavelength positional accuracy, single‐particle‐level, on‐demand quantity control, and programmable patterning capability. This direct printing approach offers a simple, flexible, accurate, and cost‐effective route to place diamond defects towards their further developments in many exciting research areas.

## 2 Results and Discussion

**Figure 1:**
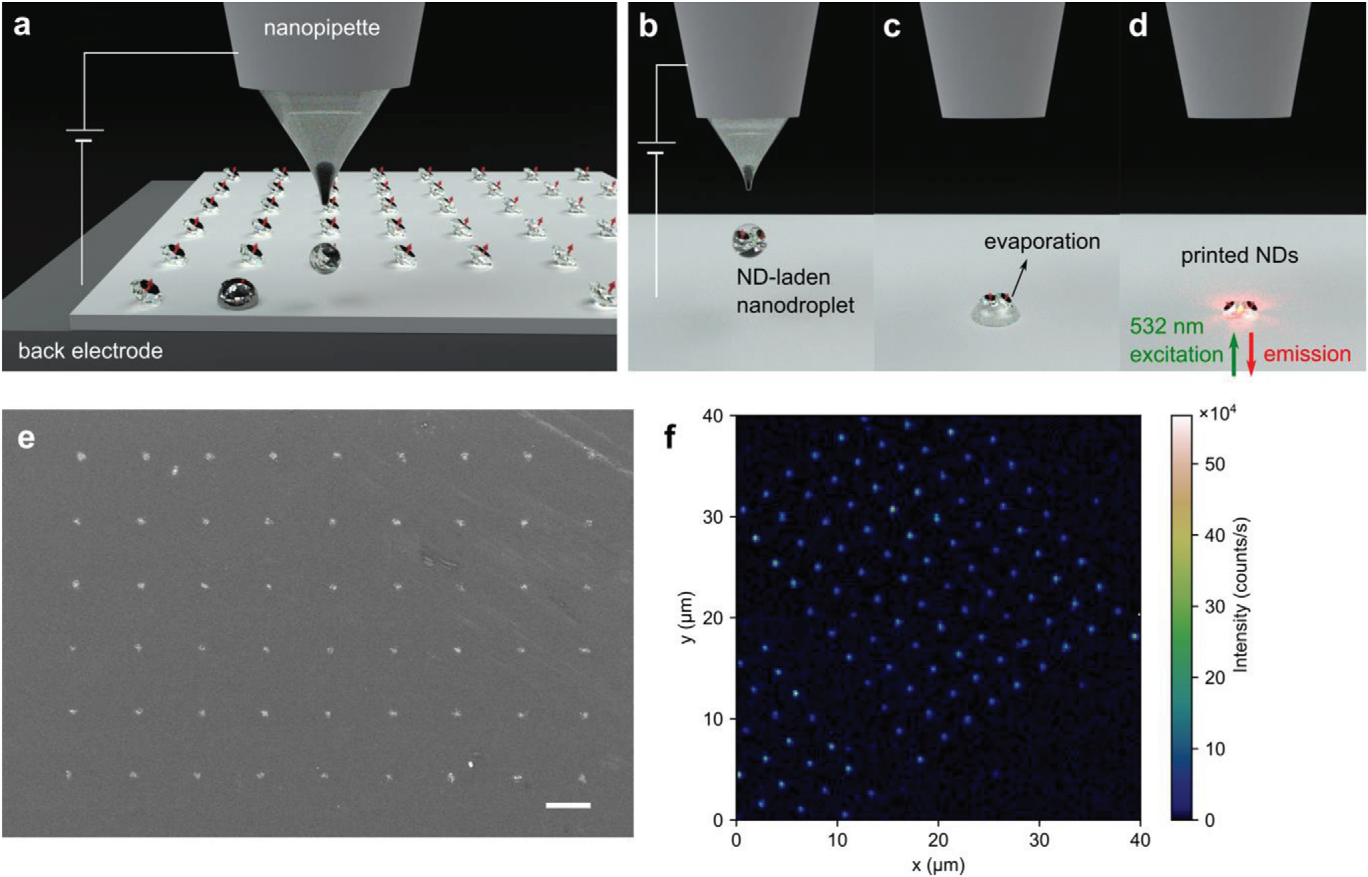
Concept of printing nanodiamonds. a) Conceptual drawing illustrating EHD printing of NV center nanodiamonds when a DC voltage is applied to a back electrode. The printing consists of three steps. b) A nanodiamonds‐laden nanodroplet is ejected upon a negative voltage of 360 V is applied to the back electrode. c) The nanodroplet is gently landed on the substrate and dried due to wetting‐enhanced solvent evaporation. d) After solvent evaporation is completed, a nanodiamonds cluster is formed and the embedded NV centers are optically accessible. e) FE‐SEM image of an array of nanodiamonds clusters with a pitch of 3 µm (scale bar: 2 µm). f) The typical confocal florescence image of the printed array of nanodiamonds clusters under 532 nm excitation (Recorded fluorescence wavelength range: 647–800 nm).

**Figure** **1**a depicts the concept of nanodiamond printing based on EHD dispensing. A glass nanopipette having an aperture diameter of 800 nm is filled with nanodiamonds suspension ink (≈40 nm sized, carboxylated surface, ≈1–4 NVs per particle) and is placed at a fixed separation of 5 µm from a substrate. The substrate is placed on a back electrode mounted at a three‐axis stepping motorized stage having sub‐µm movement precision. The use of the back electrode helps to widen the selection of substrate materials ranging from conductors, semiconductors, to insulators. When a DC pulse with programmed voltage amplitude and length is applied to the back electrode, an electrostatic attractive force is generated between the hemispherical ink meniscus and substrate, resulting in the ejection of a nanodiamonds‐laden nanodroplet with sub‐attoliter volume (Figure 1b). Once the droplet is landed on the substrate, solvent rapidly evaporates (Figure 1c), forming a nanodiamond cluster with a minute particle number. The electron spins of NV centers embedded in the printed cluster are then optically addressable under 532 nm laser excitation (Figure 1d). Note that “printing yield” is defined as the number of printed nanodiamonds clusters by one hundred attempts and used for quantitative studies in this work.

A field‐emission scanning electron microscope (FE‐SEM) image of Figure 1e shows a 9 × 6 array of nanodiamonds clusters with a pitch of 3 µm printed on a silicon substrate by applying 360 V with a pulse length of 20 ms to the back electrode for each spot. Quantitatively, the voltage amplitude higher than 350 V results in over 98% printing yield, as the applied electrostatic force overcomes the surface tension of the ink meniscus (Figure S1a, Supporting Information). Figure S1b, Supporting Information, shows an increasing trend of the printed cluster radius (marked as *R* in the inset FE‐SEM image; *R* is defined by drawing a circle along the outermost part of the nanodiamond deposits) with the voltage amplitude due to enhanced EHD ink flow and drop impact. The EHD printed nanodiamonds clusters exhibit well‐defined NV‐center fluorescence under 532 nm laser excitation. Figure 1f shows a confocal photoluminescence (PL) image of the printed cluster array exhibiting 100% fluorescence spots.

**Figure 2:**
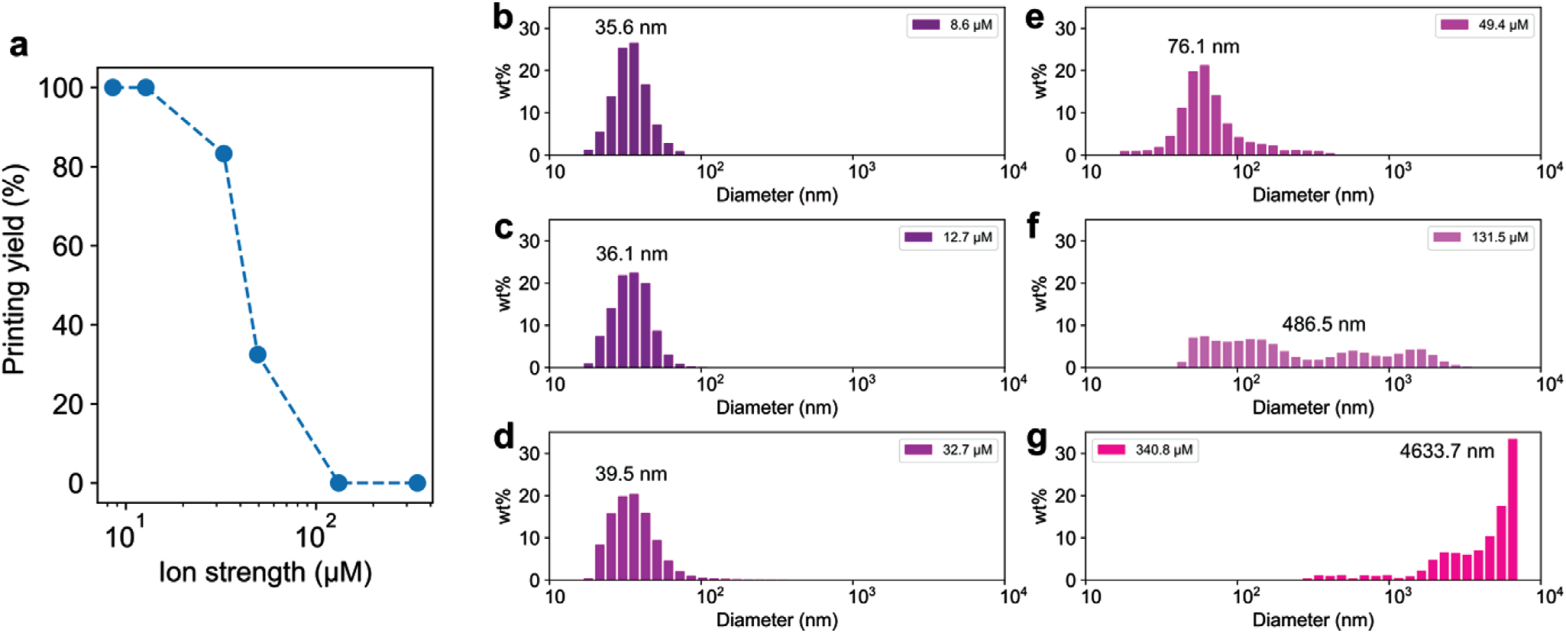
Printing yield. a) Printing yield of nanodiamonds as a function of ion strength of the suspension. b–g) Particle size distributions of the nanodiamond suspension at different ion strengths from (b) 8.6, (c) 12.7, (d) 32.7, (e) 49.4, (f) 131.5, to (g) 340.8 µm. As the ion strength increases, the printing yield decreases due to nozzle clogging by large‐sized nanodiamonds aggregates.

The printing yield—how consistent is the nanodiamond printing?—relies on the dispersion stability of the nanodiamonds ink. Uniformly dispersed ink helps to obtain a high production yield of nanodiamonds‐laden droplets. On the other hand, nanodiamond aggregates may cause clogging of the nanopipette. To achieve a satisfactory printing yield, carboxylated nanodiamonds, stabilized by electrostatic (double‐layer) repulsive forces originating from negative surface charges, are used. Besides, it is necessary to control the physical, chemical environment that can influence the dispersion stability. For example, **Figure** **2**a shows the effect of ion strength on the printing yield. At ionic strengths below 13 µm, ≈ 100% printing yields are obtained. The particle size distributions at 8.6 (Figure 2b) and 12.7 µm (Figure 2c) show the average values of 35.6 nm and 36.1 nm, respectively, similar to the single‐particle diameter of ≈ 40 nm. As the ion strength increases from 32.7, 49.4, 131.5, to 340.8 µm, the printing yield drastically decreases from 83.3%, 32.5%, 0%, to 0%, respectively (Figure 2a), due to accelerating the particle aggregation shown in Figures 2d–g. The screen of the electrostatic repulsive forces among nanodiamonds is displayed by a zeta potential decrease from −8.1 to −0.1 mV. The result indicates that stable dispersion of the ink with sufficiently low ion strength is a key requirement for reliable printing.

The number of printed nanodiamonds can be controlled at will by varying printing parameters such as ink concentration and applied pulse length. Figure S2, Supporting Information, presents the dependence of the number of nanodiamonds on ink concentration, printed by a constant electric pulse with a voltage of 360 V and a length of 20 ms. It is clear from the FE‐SEM images of printed nanodiamonds clusters (Figure S2a–c, Supporting Information) and the number distribution histograms at different ink concentrations from at 4, 2, to 1 µg mL^−1^ that ink dilution decreases the number of nanodiamonds per printed spot. The statistical mean particle numbers are 16.34 ± 4.73, 9.92 ± 3.62, and 5.02 ± 2.39 at 4, 2, and 1 µg mL^−1^, respectively.

**Figure 3:**
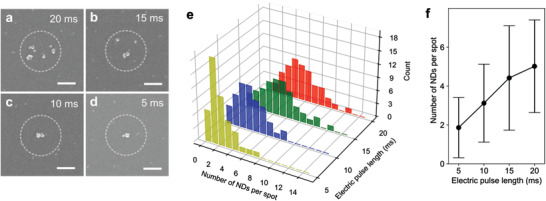
Quantity control of printed nanodiamonds per spot. a–d) FE‐SEM images of printed nanodiamonds clusters on a spot by varying electric pulse lengths from (a) 20, (b) 15, (c) 10, to (d) 5 ms, under a constant voltage of 360 V applied to the back electrode. Nanodiamonds ink with 1 µg mL^−1^ is used (scale bar: 200 nm). e) Number distribution histograms of printed nanodiamonds per spot at different pulse lengths from 20 (red), 15 (green), 10 (blue), to 5 ms (yellow). f) Statistical means of the number of printed nanodiamonds per spot at different electric pulse lengths.

A further decrease in the number of printed nanodiamonds is achieved by shortening the electric pulse length. The FE‐SEM images in **Figure** **3**a–d show a decrease in the nanodiamond number per printed spot as the 360 V pulse length shortens from 20, 15, 10, to 5 ms when 1 µg mL^−1^ ink is used. The corresponding number distribution histograms shift to decrease and become narrower as the pulse length shortens (Figure 3e). The statistical mean values of the nanodiamond numbers per printed spot are 5.02 ± 2.39, 4.42 ± 2.69, 3.12 ± 1.55, and 1.86 ± 1.55 at 20, 15, 10, and 5 ms, respectively (Figure 3f). We remark that over 38% of the printed spots by a 5 ms pulse contain a single nanodiamond, indicating that our on‐demand printing enables the single‐particle‐level control. This quantity control approach is associated with the dependence of ink ejection volume on electric pulse length. Figure S3, Supporting Information, plots a functional dependence of the printed spot radius, *r* on pulse length, *t*, corresponding to *r*(*t*) = *Kt* ^0.37±0.01^, where *K* is the proportionality constant. The growth exponent of 0.37 ± 0.01, similar to 1/3, implies that a constant flow rate is produced by a constant voltage amplitude in this experiment. The mean radius of the printed cluster by a 20 ms pulse is measured as 289.8 ± 68.9 nm, demonstrating a subwavelength emission spot size.

**Figure 4:**
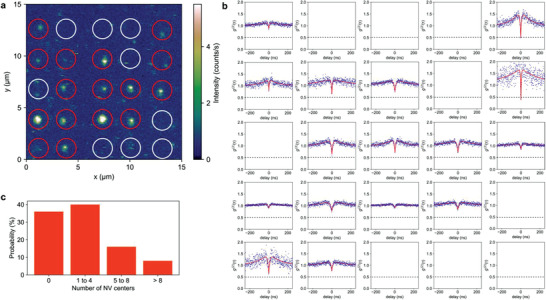
Counting the number of defects on a printed spot. a) Confocal fluorescence image of a 5 × 5 printed nanodiamonds array. Each spot is printed by a single electric pulse with a voltage amplitude of 360 V and a length of 5 ms, using 1 µg mL^−1^ nanodiamonds ink. Red circle indicates detectable NV spins whereas white circle indicates no detection. b) Measured second‐order correlation functions *g* ^(2)^(*τ*) of corresponding fluorescence spots. c) Number distribution histogram of NV centers per spot.

To prove the quantum level on‐demand printing, we perform Hanbury–Brown and Twiss (HBT) measurements and analyze the intensity‐time traces to deduce the second‐order correlation functions *g* ^(2)^(*τ*) under 532 nm laser excitation. Analyzing *g* ^(2)^(0) enables one to count the number of single emitters within a diffraction‐limited spot, according to *g* ^(2)^(0) = 1−(1/*m*), where *m* denotes the number of NV centers.^[^ ^40^ ^]^ **Figure** **4**a displays a typical confocal fluorescence image of a 5 × 5 array of 30‐nm nanodiamonds printed on a glass substrate by applying an electric pulse with a voltage amplitude of 360 V and a length of 5 ms to 1 µg mL^−1^ 30‐nm nanodiamond ink (≈1–4 NVs per particle). The printing condition chosen can deliver 1.86 ± 1.55 nanodiamonds per spot, as discussed in Figure 3. The fluorescence signals show the existence of NV centers. Figure 4b plots the corresponding *g* ^(2)^(*τ*). The measured *g* ^(2)^(0) gives a statistical result of the number of printed NV centers (Figure 4c): 40% of the printed spots contain ≈1–4 NV centers (8% of the printed spots contain only 1 NV center), 16% contain ≈5–8 NV centers, and 8% contain more than 8 NV centers. No fluorescence signal is observed from 36% of the printed spots due to the absence of NV centers at a similar rate to the statistical data of the nanodiamonds ink. We also perform the optically detected magnetic resonance (ODMR) measurement of a printed nanodiamond with a single NV center. In the absence of an external magnetic field, the observed ODMR spectrum shows a resonance frequency of ≈ 2.87 GHz which corresponds to the zero splitting of the NV center (Figure S4, Supporting Information). These results demonstrate the capability of our approach to print a small number of NV centers directly on the substrate by a single‐step process.

**Figure 5:**
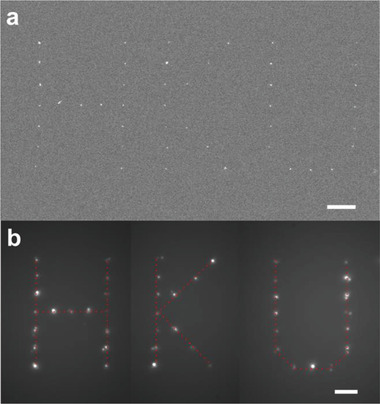
On‐demand printing of nanodiamonds containing NV centers. a) FE‐SEM image of an NV center pattern shaped with “HKU” and b) its corresponding wide‐field fluorescence image (scale bars: 4 µm).

Our maskless, open‐nanofluidic technique enables the on‐demand placement of NV‐center nanodiamonds in arbitrary patterns. **Figure** **5** shows an exemplary nanodiamonds pattern with an “HKU” logo printed on a glass substrate. The FE‐SEM image in Figure 5a shows the printing pattern consisting of nanodiamond cluster spots with a pitch of 3 µm and a positional accuracy < 127 nm. The corresponding fluorescence image (Figure 5b) confirms all the spots in the pattern contain NV centers. Although in this work we only show a few simple examples, the method could directly print various patterns made up of quantum emitters.

## 3 Conclusion

In conclusion, we have developed a direct nanoscale EHD printing that allows for the placement of nanodiamonds containing point defects at will. On‐demand control over the quantity and position of printed nanodiamonds has been demonstrated by thoroughly characterizing printing conditions as a result, the printed matter has reached the single‐particle level, containing only a few defect centers. Further research, for example, the wetting and coffee‐ring effect in nanodroplets, has the potential to improve printing precision. The method is simple and general and therefore could be extended for printing various nanodiamonds with different sizes and defect densities and types, for example, SiV^−^ centers. Furthermore, this lithography‐free approach would lower the technological barriers required for the implementation of such solid‐state quantum devices into diverse fields.

## 4 Experimental Section

### Preparation

40‐nm fluorescent nanodiamond suspension (carboxylated, ≈1–4 NV centers per particle, suspended in deionized water, purchased from Adámas Nanotechnologies) and 30‐nm fluorescent nanodiamond suspension (carboxylated, ≈1–4 NV centers per particle, suspended in deionized water, prepared by the method in previous work)^[^ ^41^, ^42^ ^]^ were used. The printing inks were prepared by diluting the nanodiamond suspensions in deionized water by a factor of 1000 and adding 0.1wt% of nonionic surfactant TX100 (Sigma Aldrich) to adjust the surface tension. For preparing a printing nozzle, a borosilicate glass nanopipette having a diameter of 800 nm was fabricated by a programmed heat‐pulling process (P‐97 Flaming/Brown Micropipette Puller, Sutter Instrument). The prepared nanopipettes, silicon substrates, and glass substrates were cleaned by rinsing with acetone, isopropyl alcohol, and deionized water under sonication for 5 min each and then by O_2_ plasma treatment for 5 min.

### EHD Printing

The printer setup consisted of a printer head and a platform. The printer head was configured with a glass nanopipette held in a three‐axis translation stage and the platform was composed of a three‐axis stepping motorized stage with a 50 nm precision (XA05A, ZA05A, Kohzu Precision), an indium tin oxide (ITO)‐coated glass plate placed on the stage as a back electrode, and a substrate on the back electrode. During EHD printing, the pipette‐substrate gap was fixed to 5 µm and programmed electric pulses with a voltage amplitude of 360 V and a length ranging from 4 s to 5 ms were applied to the back electrode using a pulse generator (NI USB‐6212, National Instruments) with an amplifier (AMJ‐2B10, Matsusada Precision Inc.). The entire EHD printing process was monitored in real‐time by using a side‐view optical microscope consisting of a long working distance objective (50×, 0.55 NA, Mitutoyo Plan Apo) and a CCD camera (DCC1545M, Thorlabs). The printing was performed under controlled relative humidity (30%) by mass flow controllers (SLA5800, brooks instrument) and controlled temperature (25 ℃) inside a custom‐made environment enclosure.

### Optical Characterizations

The characterization of NV‐center fluorescence from printed nanodiamonds was carried out using a custom‐made confocal laser scanning microscope consisting of an oil immersion objective (NA 1.45 UPLXAPO100XO), a continuous 532nm laser (300µW laser power was used during the experiment), *λ* = 647 nm long‐pass edge filter (BLP01‐647R‐25), and two single‐photon counting modules (SPCM‐AQRH‐16‐FC, Excelitas Technologies). HBT experiment was performed to characterize the number of NV centers embedded. The emission was divided by a 50/50 fiber optic coupler and collected by two single‐photon counting modules to obtain the second‐order correlation function of the time delay. The ODMR measurement (from 2.84–2.90GHz in steps of 2MHz) was performed by measuring the fluorescence intensity from the NV‐center with an integration time of 0.1 s. The wide‐field characterization was done by a custom‐made wide‐field microscope consisting of an oil immersion objective (NA 1.49 Olympus UAPON100XOTIRF), a continuous 532 nm laser, a long‐pass filter with the cut on wavelength at 647 nm, and an EMCCD camera (Evolve 512 Delta) with a tube lens at 300 mm focal length. 300 ms exposure time of the camera and the 400 mW laser power were used in the experiment.

### Material Characterizations

The exterior of printed structures was characterized by an FE‐SEM (Sigma 300, Zeiss). The particle size and Zeta potential of the nanodiamond ink were measured by using a dynamic light scattering (DLS) analyzer (Nanotrac Wave).

### Statistical Analysis

Particle size and Zeta potential were processed using DLS module and Nanotrac Wave FLEX software installed in Nanotrac Wave (Figure 2). Statistical analysis of the quantity of printed nanodiamonds clusters was manually counted and the sample size for each condition was 50 (Figure 3 and Figure S2, Supporting Information). The values represented the mean ± standard deviation of the mean. The ODMR signal of the NV centers and the corresponding *g* ^(2)^(0) value were fitted and determined by a gaussian function fit written by python (Figure 4 and Figure S4, Supporting Information). The values represented the mean ± standard error of the mean.

## Conflict of Interest

The authors declare no conflict of interest.

## Supporting information

Supporting Information

## Data Availability

Research data are not shared.
