# Supplementary material for: On‐Demand, Direct Printing of Nanodiamonds at the Quantum Level
Source: Adv Sci (Weinh). 2021 Dec 23;9(5):2103598. doi: 10.1002/advs.202103598 (PMC8844569; doi:10.1002/advs.202103598)
Supplement: Supplementary file 1 — Supporting Information [file ADVS-9-2103598-s001.pdf]

ADVANCED SCIENCE
Open Access

Supporting Information

for *Adv. Sci.*, DOI: 10.1002/advs. 202103598

On-Demand, Direct Printing of Nanodiamonds at the Quantum Level

Zhaoyi Xu, Lingzhi Wang, Xiao Huan, Heekwon Lee, Jihyuk Yang, Zhiwen Zhou, Mojun Chen, Shiqi Hu, Yu Liu, Shien-Ping Feng, Tongtong Zhang, Feng Xu, Zhiqin Chu, and Ji Tae Kim**

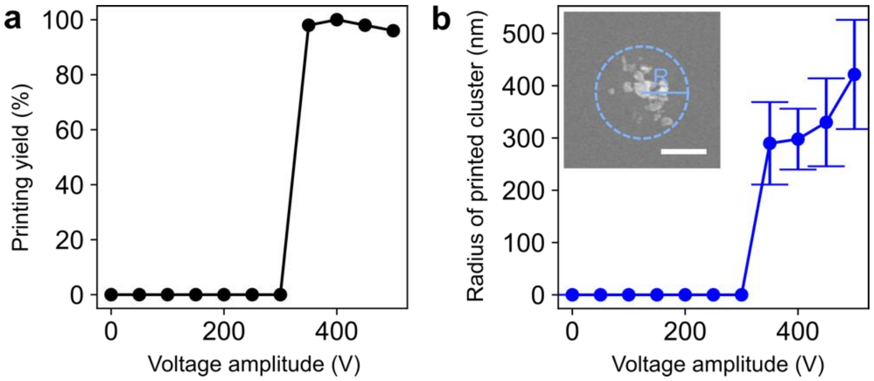

Figure S1. Voltage effect. **a)** Dependence of printing yield on voltage amplitude (pulse length: 20 ms). The voltage amplitude higher than 350 V results in over 98% printing yield. The onset voltage, $V_{\text{onset}} \approx 341\,V$, is calculated from $V_{\text{onset}} \approx \sqrt{\frac{2\gamma R\cos\theta}{\varepsilon_0}}\ln\left(\frac{4l}{R}\right)$, where $\gamma = 30\,mN/m$ is the surface tension of the ink, $R = 0.5\,\mu m$ is the nozzle radius, $\theta = 49.3°$ is the con semi vertical angle, $\varepsilon_0 = 8.85 \times 10^{-12}\,C^2/N \cdot m^2$ is the permittivity of free space, $l = 175\,\mu m$ is the nozzle-electrode plate distance. **b)** Dependence of printed spot radius on voltage amplitude. Inset: FE-SEM image of a printed nanodiamonds cluster spot by applying a voltage of 360 V with a pulse length of 20 ms. (scale bar: 200 nm).

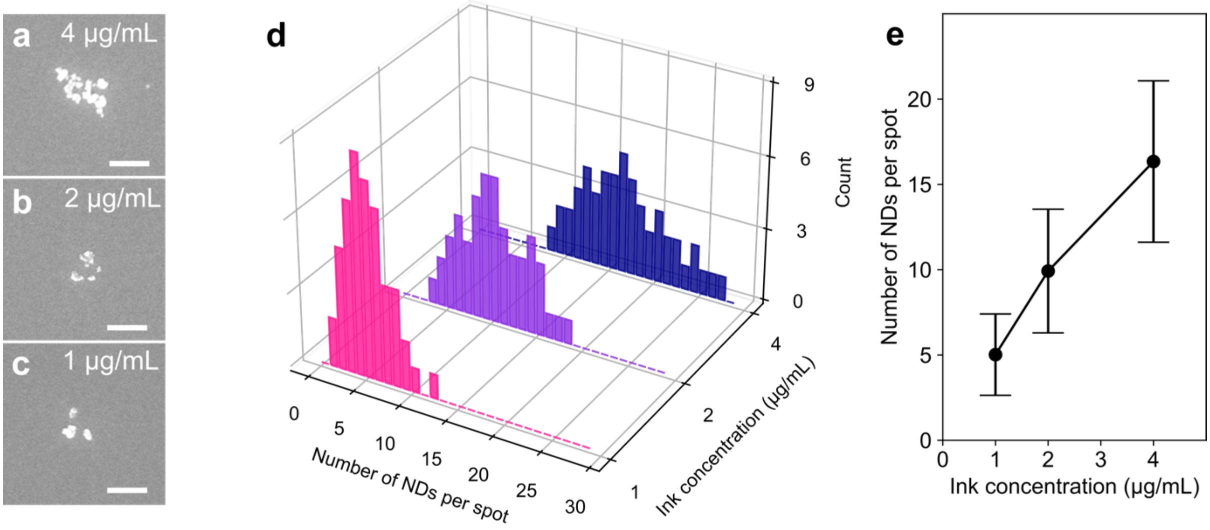

Figure S2. Ink concentration effect. **(a-c)** FE-SEM images of printed nanodiamonds clusters on a spot by varying concentration of nanodiamonds ink from **a)** 4, **b)** 2, to **c)** 1 µg/mL. Each spot is printed by a single electric pulse with a voltage amplitude of 360 V and a length of 20 ms (scale bar: 200 nm). **d)** Number distribution histograms of printed nanodiamonds per spot at different ink concentrations from 4 (dark blue), 2 (violet), to 1 µg/mL (pink). **e)** Statistical means of number of printed nanodiamonds per spot at different ink concentrations.

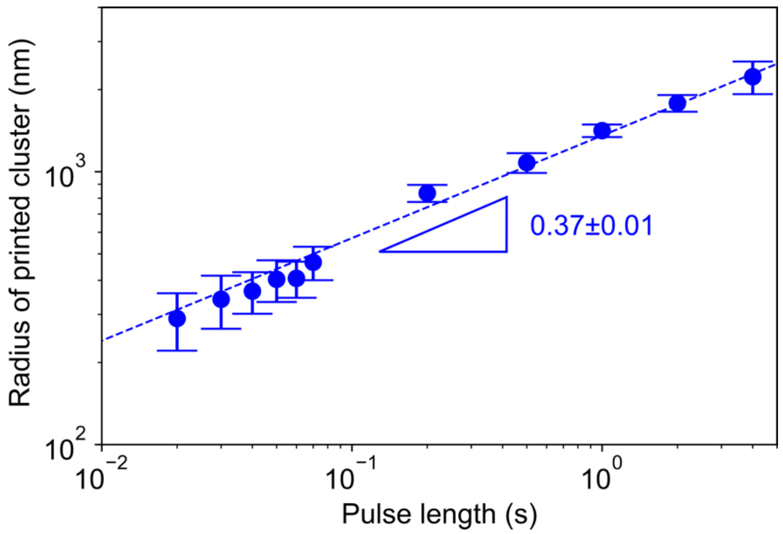

Figure S3. Radius of printed nanodiamonds spot, r versus pulse length, t. The dashed line corresponds to a functional dependence $r\ (t) = (1357 \pm 21)\ t^{\,0.37\pm0.01}$.

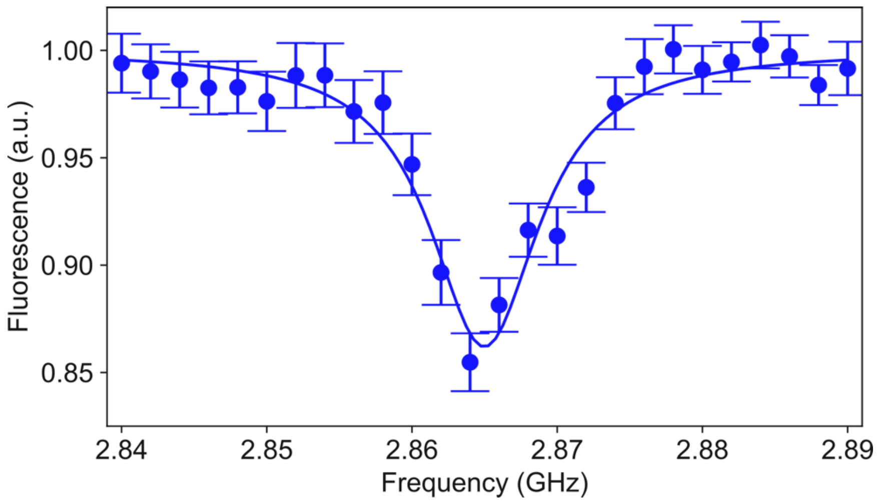

Figure S4. ODMR signal of a single NV center in a single nanodiamond without an external magnetic field. The observed resonance frequency corresponds to the zero field splitting of the NV center.
